# Biases in grant proposal success rates, funding rates and award sizes affect the geographical distribution of funding for biomedical research

**DOI:** 10.7717/peerj.1917

**Published:** 2016-04-11

**Authors:** Wayne P. Wahls

**Affiliations:** Department of Biochemistry and Molecular Biology, University of Arkansas for Medical Sciences, Little Rock, AR, United States of America

**Keywords:** Biomedical research, Federal funding, Science policy

## Abstract

The ability of the United States to most efficiently make breakthroughs on the biology, diagnosis and treatment of human diseases requires that physicians and scientists in each state have equal access to federal research grants and grant dollars. However, despite legislative and administrative efforts to ensure equal access, the majority of funding for biomedical research is concentrated in a minority of states. To gain insight into the causes of such disparity, funding metrics were examined for all NIH research project grants (RPGs) from 2004 to 2013. State-by-state differences in per application success rates, per investigator funding rates, and average award size each contributed significantly to vast disparities (greater than 100-fold range) in per capita RPG funding to individual states. To the extent tested, there was no significant association overall between scientific productivity and per capita funding, suggesting that the unbalanced allocation of funding is unrelated to the quality of scientists in each state. These findings reveal key sources of bias in, and new insight into the accuracy of, the funding process. They also support evidence-based recommendations for how the NIH could better utilize the scientific talent and capacity that is present throughout the United States.

## Introduction

The National Institutes of Health (NIH) is the federal steward of biomedical research in the United States. Ensuring equal access to research grants and grant dollars, geographically, is a fundamental tenant of the NIH’s mission to manage a diverse, robust and sustainable research ecosystem that maximizes return on taxpayers’ investments (Lorsch, 2015).

One way to help achieve that goal has its origins in 1950’s legislation that created the National Science Foundation (NSF) (National Science Foundation Act, 1950). The congressional mandate included “to strengthen basic research and education in the sciences...throughout the United States...*and to avoid undue concentration of such research and education*” (emphasis added). Creation of the Experimental Program to Stimulate Competitive Research (EPSCoR) in 1979 extended this mandate and increased support to disadvantaged states that were receiving a relatively small share of NSF funds (EPSCoR/IDeA Foundation, 2015).

The general objective of the NSF EPSCoR program, to help address geographical disparities in federal support for research, was subsequently adopted by the Department of Energy, the Department of Commerce, the Department of Defense, the Department of Agriculture, the Environmental Protection Agency, the National Aeronautics and Space Administration, and the National Institutes of Health (EPSCoR/IDeA Foundation, 2015). The NIH’s EPSCoR-type program, the Institutional Development Award (IDeA) program, is by far the largest of the EPSCoR-type federal programs ($273 million in fiscal year 2015) (EPSCoR/IDeA Foundation, 2015; National Institute of General Medical Sciences, 2015). Its specific goals, laid out succinctly at implementation of the program, are to enhance the ability of institutions and investigators in disadvantaged states to compete successfully for NIH-funded grants (based on metrics such as grant application success rates and total funding) (National Institutes of Health, 1993).

In 2010 (signed into law in 2011), Congress charged the NSF Director to “contract with the National Academy of Sciences to conduct a study on all Federal agencies that administer an EPSCoR or a program similar to the EPSCoR” (America COMPETES Reauthorization Act, 2010). The National Academies Press subsequently published a multi-agency committee report evaluating the federal EPSCoR-type programs since their inception (Committee to Evaluate the Experimental Program to Stimulate Competitive Research et al., 2013).

Three of the committee’s findings stand out. First, the talent necessary to succeed in science resides in all states. Second, the programs have enhanced the nation’s human capital by strengthening research infrastructure and training in states that were disadvantaged prior to the program’s arrival. Third, the aggregate share of federal grant funding to eligible states has not changed significantly over the course of the programs. Similarly, the success rates of grant applications from program-targeted states have remained consistently lower than those of other states since program inception. In other words, neither the congressional intent nor specific program goals have been fully realized.

Why have laudable, beneficial programs supported by legislative mandate and with clearly defined objectives not achieved their specific goals? To gain insight into potential causes of geographical disparities in funding for biomedical research and why they have persisted, funding metrics were examined for all NIH research project grants (RPGs) over a ten year period (2004–2013).

## Materials and Methods

### Data sets

Publicly available data were compiled in, and additional new data sets were derived using, Excel for Mac V12.3.6 (Microsoft Corp., Redmond, WA, USA). Data compiled by state (for individual years and for multiyear means presented in the paper) are available in Data S1. State population data were obtained from the United States Census Bureau (2013). Data on grant application success rates and investigator funding rates were obtained through a Freedom of Information Act request (FOI Case No. 42901) to the NIH Office of Extramural Research (National Institutes of Health, 2014). Values are total rates for all types of applications (new, renewal and revision). Data on number of RPG awards and total RPG funding to each state were obtained by searching the NIH Research Portfolio Online Reporting Tool (RePORT) (National Institutes of Health, 2015). Search parameters were fiscal year (individually 2004–2013) and funding mechanism (research project grants); outputs were data and visualize (by state), exported to Excel. Data on publication rates were obtained, first, by using the “awards by location” quick-link tab of RePORT (National Institutes of Health, 2015) to obtain total RPG funding and lists of RPG project numbers, by state, for 2011. Second, the RPG project numbers of each state were used to search PubMed (National Center for Biotechnology Information, 2014) for RPG-supported publications in 2011–2013. The number of publications from each state citing the 2011 project numbers was normalized to total funding in that year.

### Statistics

Data set comparisons, statistical tests, regressions and plots were generated in Prism for Mac V5.0b (GraphPad Software, Inc., La Jolla, CA, USA) using default settings. Analyses of data sets binned by per capita funding quartile were conducted using the Kruskal-Wallis Test with post hoc testing by the Dunn’s Multiple Comparison Test. The *p* values of the former are from guassian approximation; those of the latter are adjusted. Standard linear regression was used to test for associations between both non-transformed and log-transformed data sets. Similar results and identical conclusions were obtained with each approach: analyses of non-transformed data are presented below, those of log-transformed data are in Supplemental Information 1.

### Ethics Statement

It was not considered necessary to submit this study for ethical review given the nature of the project, which involved analyses of population data by state without any identifying information for individuals or institutions.

## Results

Analyses NIH RPG funding levels, grant proposal success rates, investigator funding rates, and RPG-supported publications of individual states revealed the following.

**Table 1:** Per capita research project grant funding by state.

Rank	State	Per capita funding
1	Massachusetts	$283.00
2	District of Columbia	$233.11
3	Maryland	$135.86
4	Connecticut	$103.59
5	Rhode Island	$101.95
6	Washington	$96.65
7	Pennsylvania	$85.00
8	New York	$81.22
9	North Carolina	$79.12
10	Vermont	$75.24
11	California	$67.91
12	Minnesota	$67.61
13	Oregon	$58.99
14	New Hampshire	$57.85
15	Missouri	$54.62
	*Nation*	*$53.68*
16	Tennessee	$52.08
17	Wisconsin	$51.31
18	Iowa	$50.99
19	Colorado	$48.87
20	Michigan	$46.89
21	Utah	$46.64
22	Ohio	$45.21
23	Illinois	$44.44
24	Alabama	$35.61
25	Nebraska	$34.64
26	Georgia	$32.59
27	Texas	$32.50
28	New Mexico	$30.77
29	Virginia	$29.69
30	Kentucky	$27.84
31	Maine	$26.85
32	Indiana	$26.73
33	Hawaii	$24.59
34	New Jersey	$22.32
35	Arizona	$22.00
36	Kansas	$20.94
37	South Carolina	$20.53
38	Louisiana	$19.80
39	Montana	$19.70
40	Delaware	$19.04
41	Florida	$17.59
42	Arkansas	$16.36
43	Oklahoma	$15.09
44	North Dakota	$9.73
45	South Dakota	$9.26
46	Mississippi	$7.28
47	West Virginia	$7.03
48	Wyoming	$5.40
49	Nevada	$4.99
50	Alaska	$4.94
51	Idaho	$2.36
52	Puerto Rico	$2.30

**Notes.**

Data are mean of values for fiscal years 2004–2013. The national per capita value is included (*Nation*); background shading groups states by funding quartile.

### Scope and magnitude of disparity

**Figure 1:**
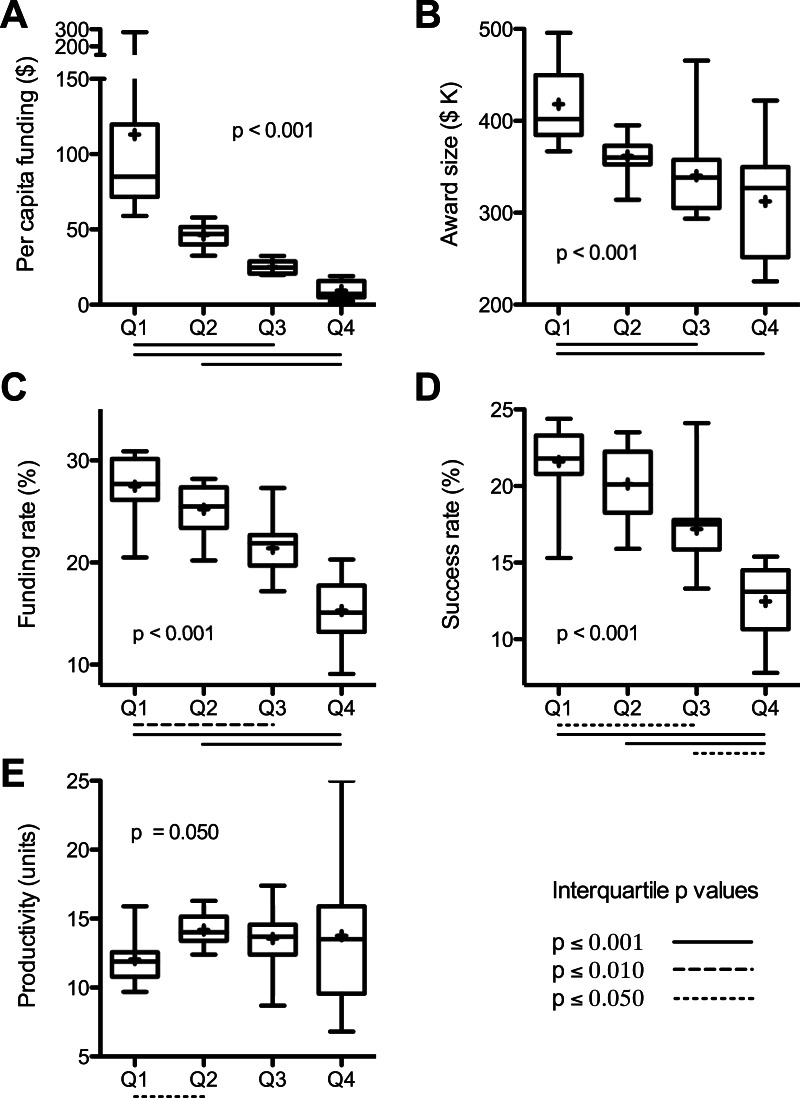
Interquartile analyses of data sets. Values from Table S1 were binned by per capita funding quartile. Box and whisker plots (median and quartile ranges) and mean values (+) are for: (A) per capita funding; (B) average award size; (C) per investigator funding rate; (D) per application success rate; and (E) scientific productivity. Overall probability values (inset) are from Kruskal-Wallis Test; adjusted *p* values below *X* axis are for significant interquartile differences by Dunn’s Post Test. Values are means of 2004–2013 data except productivity, which are sum of 2011–2013 publications citing 2011 awards normalized to funding in that year.

There was a greater than 100-fold range in the annual per capita research project grant (RPG) funding from the NIH to individual states, Washington, D.C. and Puerto Rico (“states”) (Table 1) (United States Census Bureau, 2013; National Institutes of Health, 2015). The top ten states were awarded, on average, nineteen times more RPG funding per capita than the bottom ten states. Moreover, the distribution of funding relative to the national per capita value was lopsided: Fifteen states (29%) were overfunded and thirty-seven states (71%) were underfunded. If one considers the data by funding rank quartile (Fig. 1 and Data S1), nearly two thirds of all RPG dollars were allocated to one quarter of the states. Such disparities have existed for decades and have persisted despite best intentions of the IDeA program (Committee to Evaluate the Experimental Program to Stimulate Competitive Research et al., 2013; National Institutes of Health, 2015).

### Sources of disparity

**Figure 2:**
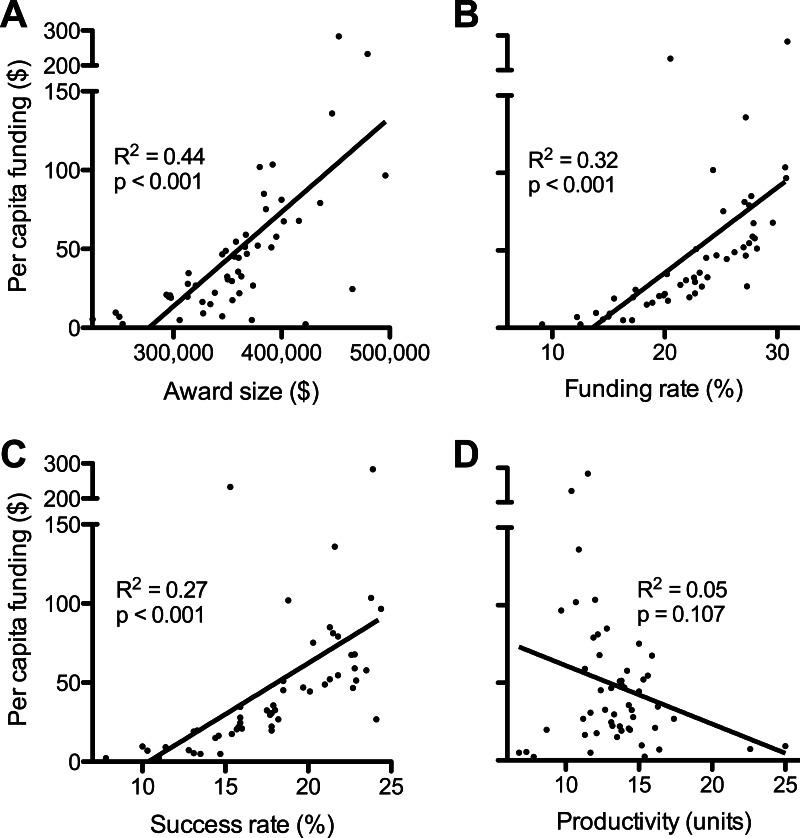
Factors affecting disparities in funding. Plots show linear regressions of state per capita RPG funding as a function of: (A) average award size; (B) per investigator funding rate; (C) per application success rate; and (D) scientific productivity. Values are as described in Fig. 1 and numerical values by state can be found in Data S1. Regression analyses of log-transformed data are in Supplemental Information 1.

One factor contributing to the state-by-state funding disparities can be found in the sizes of RPG awards (National Institutes of Health, 2015). There was a positive correlation between average funds per RPG by state and the per capita funding levels (Fig. 2A). Investigators in the top quartile (per capita funding) of states each received, on average, about $106,000 more per RPG each year than investigators in the bottom quartile of states (Fig. 1B). (Average award size is affected by the sizes of individual awards and by how different types of RPGs are distributed. For example, from 2004 to 2013 the NIH allocated 64% of high value P01 RPGs to the top quartile of states, whereas the bottom quartile of states received only 3% of P01 RPGs.)

Another contributing factor, notwithstanding its genesis, is demographics. States that have a higher population density of scientists applying for RPGs would be expected to secure a disproportionate share of grant dollars. This can be taken into account by using the NIH “funding rate” statistic, which is the fraction of applicants that are funded in a given year (Rockey, 2014). The funding rates (National Institutes of Health, 2014) were not equivalent between states but were instead spread over a broad range, which deviates from predictions of the simple demographics model. On average, investigators in the top quartile states were about twice as likely to get funded as those in the bottom quartile of states (28% vs. 15%) (Fig. 1C). Overall, there was a positive correlation between state funding rates and per capita funding levels (Fig. 2B).

Similarly, one can account for the number of RPG applications from each state by using the NIH “success rate” statistic, which is the percentage of applications that receive funding (Rockey, 2014). The success rates (National Institutes of Health, 2014) were not equivalent between states and there was a positive correlation between success rates and per capita funding (Fig. 2C). Individual grant applications from investigators in the top quartile of states were, on average, about twice as likely to be funded as applications from investigators in the bottom quartile of states (22% vs. 12%) (Fig. 1D).

Higher per application success rates, higher per investigator funding rates, and larger award sizes each contribute to the overfunding of some states; whereas lower success rates, lower funding rates, and smaller award sizes contribute to the underfunding of other states.

### Relationship between disparity and productivity

Scientific merit review (peer review) and administrative review rank order grant applications and make funding decisions, respectively. Thus, differences in the priority ranking of applications at the time of scientific merit review drive, in part, the disproportionate allocation of research dollars to individual states. What actually dictates the differences in success and funding rates between states? Is it possible that investigators in funding-rich states are simply “better” scientists than those of funding-poor states, and hence compete more effectively for funding?

The scientific productivity of RPG-funded investigators, as measured by the number of grant-supported publications per grant dollar awarded to each state (National Center for Biotechnology Information, 2014; National Institutes of Health, 2015), provides an objective metric for the quality of scientists in each state. An obvious caveat is that publication rate is but one many possible metrics for gauging the productivity or “quality” of investigators (see ‘Discussion’). Overall, there was no significant association between productivity and per capita funding (Fig. 2D). Moreover, the average productivities in the lesser-funded three quartiles of states were each higher than that of the top-funded quartile of states (Fig. 1E). This is remarkable given that scientists in funding-poor states are handicapped by lower success rates and smaller award sizes and, presumably, by more frequent and longer interruptions of funding, as well as by less extensive scientific infrastructure (a product of chronic funding disparities).

## Discussion

This study revealed that geographical biases in the way that NIH RPG funding is allocated contribute significantly to vast disparities (> 100-fold range per capita) in the amount of funding to each state (Fig. 2, Table 1). The findings provide an explanation for why legislative and administrative efforts to address such disparities have been ineffective. They also support evidence-based recommendations for how the NIH could better utilize the scientific talent and capacity that is present throughout the United States. Elaboration is provided below.

### Relevance of disparity to grant proposal scores being poor predictors of outcome

Publication counts provide a primary measure of scientific productivity and are used by NIH officials to analyze things like return on taxpayers’ investments in research and to optimize funding allocations (e.g., Berg, 2012; Lorsch, 2015). The state values tabulated in this study were normally distributed and most were clustered near the mean (Supplemental Information 1). This is consistent with previous findings that each of the United States contains the talent to carry out research (Committee to Evaluate the Experimental Program to Stimulate Competitive Research et al., 2013). Notably, there was no significant association overall between the normalized publication counts and allocations of funding (Fig. 2D). Thus, at least by this metric, state-by-state differences in productivity cannot explain why funding-rich states have higher success rates, higher funding rates, and higher per RPG funding levels than funding-poor states.

Michael Lauer (NIH Deputy Director for Extramural Research) has come to similar conclusions using different metrics: Projects funded by the National Heart, Lung and Blood Institute (NHLBI) with the poorest priority scores from reviewers produced as many publications, and had as high a citation impact per grant dollar spent, as projects with the best priority scores (Danthi et al., 2014). This occurred even though the low-rated investigators were awarded less money than their top-rated peers. Similarly, there was no significant association between priority scores assigned during peer review and time to publication of completed cardiovascular trials (Gordon & Lauer, 2013).

Congruent findings have been made for research supported by other NIH institutes and federal agencies. Further analyses of NHLBI award data (Kaltman et al., 2014; Lauer et al., 2015), and of data from the National Institute of General Medical Sciences (NIGMS) (Berg, 2012), the National Institute of Mental Health (NIMH) (Doyle et al., 2015), and the NSF (Scheiner & Bouchie, 2013) have each revealed little or no association between grant proposal scores and scientific outcomes of funded proposals. To the extent tested the findings apply for multiple measures of outcome (e.g., publication rate, number of highly cited publications, overall citation impact), even after accounting for additional variables. (A cogent description of parameters affecting the interpretation of bibliometric data as indicators of scientific productivity, along with analogies understandable by a lay audience, can be found in Lauer et al. (2015). For example, a modest association between grant percentile score and scientific outcome, as assessed by highly cited publications per grant, disappears when adjusted for award size.)

These findings, from multiple studies, indicate that the peer review process is not effectively predicting outcomes: review panels are unable to accurately rank the relative quality of investigators and projects (Mervis, 2014). Randomness (imprecision) intrinsic to the peer-review process and implicit biases (subconscious positive or negative attitudes) have each been implicated (Ginther et al., 2011; Graves, Barnett & Clarke, 2011; Moss-Racusin et al., 2012; Lai, Hoffman & Nosek, 2013). Since NIH program officials rely heavily on rank-ordered priority scores for funding decisions, it is crucial to detect and correct the sources of variance and bias.

Toward this end, analyses of NIH-wide RPG data revealed specific sources of bias that are seemingly unrelated to the productivity of investigators (Fig. 2). Statistically significant biases in success and funding rates of RPG applications from different states and in the average amount of funds allocated per RPG each contribute to the vast differences in NIH funding per capita to individual states (Table 1). Because these biases are quantifiable, they should be correctable (recommendations below).

### Good IDeAs unfulfilled

The findings also provide a simple explanation for why legislative and administrative efforts to promote a more equitable distribution of funding geographically (e.g., National Science Foundation Act, 1950; National Institutes of Health, 1993; EPSCoR/IDeA Foundation, 2015; National Institute of General Medical Sciences, 2015) have been ineffective (Committee to Evaluate the Experimental Program to Stimulate Competitive Research et al., 2013): The IDeA program (and other EPSCoR-type programs) does not directly address proximate causes of geographical funding disparities, namely, the biases in grant proposal scoring and award size (Fig. 2). Moreover, potential benefits of the IDeA program are restricted by its level of funding (less than 1% of the NIH budget), which is set by Congress (EPSCoR/IDeA Foundation, 2015). This amount of funding for remediation (millions of dollars) pales in comparison to the magnitude of geographical funding disparities (billions) (Supplemental Information 1). The program is a good idea with clear benefits (Committee to Evaluate the Experimental Program to Stimulate Competitive Research et al., 2013), but its focus and funding each prevent it from having more than an incremental impact on geographical funding disparities.

### Potential solutions to bias

The geographical success rate bias (which underlies funding rate bias) should be corrected because it denies funding to otherwise meritorious research projects, affects adversely the majority of states’ scientific infrastructure (including education), and undermines the broader objectives of the NIH. (Additional impacts are described in Supplemental Information 1.) The bias in funds allocated per RPG should also be addressed.

Part of the solution is manifest, simple and equitable. As early as the next cycle of administrative review, the NIH could eliminate the significant bias in success rates between states. Implementation would be facile. For example, if the national success rate for R01 applications is 18%, the NIH would fund 18% of R01 applications from each state.

Establishing parity of success rates between states would affect neither past funding imbalances nor the scientific merit review of future applications. It would simply adjust for biases (implicit or explicit) about the overall quality of scientists in each state and would help to level the field moving forward.

Eliminating the significant bias in average funds allocated per RPG would also seem straightforward. The NIH could adjust the budgets of individual grants (which is done routinely) to establish interstate parity of average award size. Importantly, the individual award sizes within each type of RPG and within each state could still vary substantially, preserving flexibility and power of the funding system. Notably, this more equitable distribution of funds could be implemented incrementally without altering the budgets of any active grants. The process could be completed by the time all current RPGs end or are renewed competitively.

The proposed remedies to bias (above) are simple, direct and would be effective, but might raise questions about whether a quota-based approach is the best solution. One might propose, instead, that the NIH could develop a more equitable distribution system that promotes geographical diversity without specific quotas. Indeed, this was the intent of the IDeA program (extant since 1993) (National Institutes of Health, 1993; National Institute of General Medical Sciences, 2015). But while that approach has strengthened institutional research infrastructure and training of junior investigators, it has had no obvious impact on relative success rates of applications from or the share of NIH funding to disadvantaged states (Committee to Evaluate the Experimental Program to Stimulate Competitive Research et al., 2013). This demonstrates empirically a crucial point. Indirect approaches, in particular those that are underfunded, are unlikely to have any meaningful impact upon geographical biases in success rates, funding rates and award sizes.

It should be emphasized that eliminating bias would not eliminate geographical disparities in funding. This is because the disparities stem as much from demographics as from bias (Supplemental Information 1). Under conditions of equal access (absence of bias), states with higher population densities of applicants would continue to secure a disproportionate share of RPG dollars. The United States would still be a country in which the majority of funding for biomedical research is concentrated in a minority of states.

### Concluding perspectives

Jon Lorsch, Director of the NIGMS, has pointed out that “it is impossible to know where or when the next big advances (in biomedical research) will arise, and history tells us that they frequently spring from unexpected sources” (Lorsch, 2015). Every state in the union contains the talent and capacity to carry out research (Committee to Evaluate the Experimental Program to Stimulate Competitive Research et al., 2013); and the ability of our nation to most efficiently make breakthroughs on the biology, diagnosis and treatment of human diseases requires that physicians and scientists in each state have equal access to research grants and grant dollars. Now, decades after NIH officials and members of Congress recognized the need to address geographical funding disparities (e.g., National Institutes of Health, 1993), it is time address underlying causes of the problem.

### Addendum: limitations and future directions

Although this study identified three types of bias that contribute to geographical funding disparities; the success rate, funding rate and award size data provide no insight into the proportional contributions of scientific merit review and administrative review. Future analyses of grant application priority score distributions, of applications being funded or denied funding out of priority score order, and of administrative changes to budgets by state, might be informative.

The current data also provide no insight into award parameter differentials at the level of individual investigators or institutions, which presumably contribute to the state-level biases and hence would be worth studying in the future.

The metric for productivity that was employed, grant-supported publications, is of limited scope and duration. Analyses of secondary metrics such as impact factors of journals in which the publications appear, tertiary metrics such as field-normalized citation counts, and additional factors such as the types of research conducted might be worthwhile, but would not affect conclusions stated in the title. One must also consider the possibility that the kinds of bias that affect grant application success rates from different states also affect journals’ decisions on which manuscripts to publish and authors’ decisions on what papers to cite—each of which affects citation-based metrics. An equally challenging issue is that the historical and current funding differentials affect nearly every aspect of what scientists need to do their jobs effectively. Examples include the amount of funding for their own research, the constellation of funded investigators with whom they interact locally, bricks and mortar, administrative support, core facilities and instrumentation. Thus, unless and until one can account quantitatively for impacts of geographical bias and geographical funding differentials upon various measures of productivity (e.g., bibliometrics), such measures should be interpreted with caution. Within that context, the current data on productivity are consistent with the null hypothesis—that every state contains the talent to carry out biomedical research.

## Supplemental Information

10.7717/peerj.1917/supp-1 Data S1 Table S1. Summary of research project grant characteristics by location. Table S2. Per capita funding of research project grants by location. Table S3. Average sizes of research project grants by location. Table S4. Funding rates of research project grants by location. Table S5. Success rates of research project grants by location. Table S6. Productivity values of research project grants by location.

10.7717/peerj.1917/supp-2 Supplemental Information 1 Supplemental Information

## Additional Information and Declarations

### Data Availability

The following information was supplied regarding data availability: All data are contained in the manuscript and in the Supplemental Information.
