# Supplementary material for: Biases in grant proposal success rates, funding rates and award sizes affect the geographical distribution of funding for biomedical research
Source: PeerJ. 2016 Apr 11;4:e1917. doi: 10.7717/peerj.1917 (PMC4830231; doi:10.7717/peerj.1917)
Supplement: Supplemental Information 1 [file peerj-04-1917-s002.pdf]

Biases in grant proposal success rates, funding rates and award sizes affect the geographical distribution of funding for biomedical research

Wayne P. Wahls

Department of Biochemistry and Molecular Biology, University of Arkansas for Medical Sciences, Little Rock, Arkansas, United States of America

E-mail: wahlswaynep@uams.edu

Each of the United States contains the talent and capacity to carry out biomedical research, but much of this talent and capacity is underutilized due to disparities in the allocation of research grant dollars to individual states.

I reside in, and compete for NIH grant funding from within, the state of Arkansas. I therefore use the Natural State as an example for how the distribution of funding to individual states affects local economies, as well as the diversity and productivity of our national biomedical research enterprise.

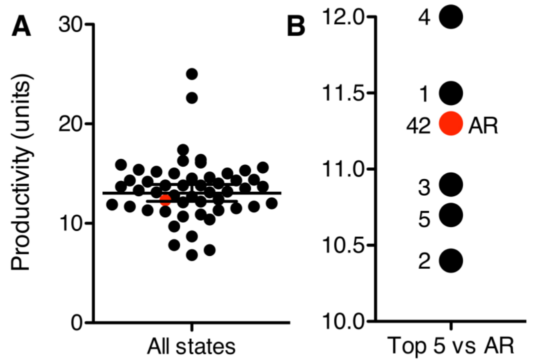

Figure S1. Productivity values are normally distributed and most are clustered near the mean. Scatter plots show productivity values of each state; lines indicate weighted mean and 95% confidence interval. Data are for: (A) all states; and (B) author's state compared to the five top-funded states (number indicates per capita funding rank).

Distribution of scientific talent and capacity

The per capita RPG funding to individual states varied over a broad range (>100-fold) (**Table 1**). In contrast, the scientific productivity values were normally distributed and most were tightly clustered around the mean (**Figure S1A**). Interestingly, the productivity value of my under-funded state, Arkansas (42nd in per capita funding), was close to the national mean and compared favorably to those of the top-funded states (**Figure S1B**). Notwithstanding this comparison, the clustering of values is consistent with previous findings that each of the United States contains the talent to carry out research (*Committee to Evaluate the Experimental Program to Stimulate Competitive Research, 2013*).

While there was no significant association between investigator productivity and per capita funding overall, several top-funded states had productivity values below the mean (**Figure 2D**).

To gain further insight into the differences, the data were binned into quartiles (by per capita funding) for further analyses. The mean productivity in each of the bottom three quartiles of states was higher or significantly higher than that of the top-funded quartile (**Figure 1E**). This suggests that investigators in top-funded states are, on average, less productive (per dollar of RPG funding) than those in lesser-funded states. It will be interesting to see if this finding is confirmed by analyses of additional data sets and using additional metrics for productivity. For instance, predoctoral and postdoctoral fellowships and training grants (examples of NIH funding not in the RPG category) are each allocated preferentially to the top-funded quartile of states (*National Institutes of Health, 2015*) and those dollars might be included in the denominator for calculations of productivity.

Each of the United States also has the capacity to carry out research. The NIH receives far more grant applications than its budget can support and only a small fraction of proposals get funded each year (success rate) (*Rockey, 2014; National Institutes of Health, 2014*). Less than one quarter of investigators who apply for RPG funding each year get funded (funding rate) (*Rockey, 2014; National Institutes of Health, 2014*), which provides a measure of scientific capacity that is being utilized. More than three-quarters of the applicants do not get funded and this unutilized capacity can be found in every state. However, the under-funded states have a greater proportion of unutilized capacity than over-funded states because the funding rates of disadvantaged states are significantly lower than those of advantaged states (**Figure 1C**).

There is, to my knowledge, no reason to believe that ensuring equal access to research grants and grant dollars (absence of geographical bias) would affect the quality, significance or innovation of proposals from advantaged and disadvantaged states. Closing the success rate and funding rate gaps between states would involve only minor changes to unutilized capacity. The vast majority of proposals from each state would still not get funded, so competition would remain intense. Only the best scoring, most meritorious proposals from each state would be funded—and scientists in each state would compete on equal footing for this grant support.

Economic impacts

Taxpayers in each of the United States send revenue to the Federal government and a portion of that revenue is allocated to the NIH. The NIH uses some of its funds for facilities and administration and some to support intramural research, but the bulk of the NIH budget is allocated back to individual states (*Department of Health and Human Services, 2015*). The majority of this extramural funding (53% of the total NIH budget in fiscal year 2015) is used to support RPGs, which are the subject of this study.

How much does each state contribute to the extramural RPG funding pool? For any given state the amount can be estimated by multiplying that state's population by the national per capita RPG funding level ($53.68, mean of data for 2004 to 2013). For the state of Arkansas (mean population of 2,874,258), this amounted to $154 million per year. Such estimates are imprecise because the per capita Federal tax burden varies between states (e.g., ours is higher than the national average) (*Internal Revenue Service, 2015*), but the values are adequate for the sake of illustration.

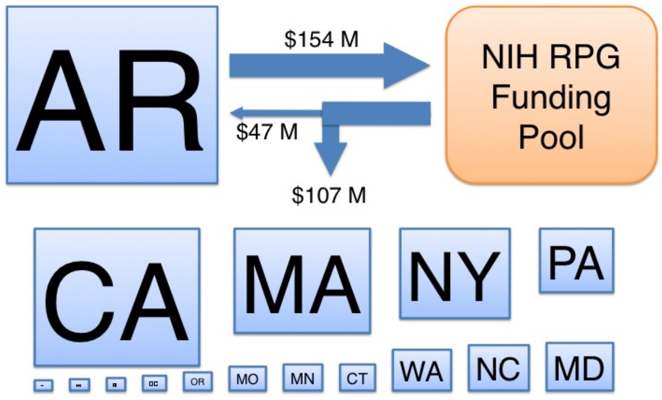

Figure S2. Flux of research project grant dollars. Example illustrates the flow of tax dollars from an individual state into the extramural RPG funding pool and then back to states. The font size of each state abbreviation is proportional to the number of Arkansas-derived dollars secured by each state. Dollar values into the funding pool and to other states are estimates (see text for additional details).

Because each state contains the talent and capacity to carry out biomedical research, one might expect that the amount of RPG funding from the NIH to each state would be similar to the amount of money that each state contributes to the NIH RPG funding pool. However this is not the case, as exemplified by funding to Arkansas. For every estimated tax dollar that I contributed to the RPG funding pool from 2004 to 2013, only about 30 cents came back to support research in Arkansas (**Figure S2**). The other ~70 cents went to subsidize research in the 15 states that were over funded (**Table 1**).

The state populations and RPG funding levels also allow one to determine the flux of dollars between each of the states. The ultimate destinations of Arkansas RPG tax dollars are shown in **Figure S2**, with font sizes of state abbreviations being proportional to the number of dollars that originated from Arkansas. Arkansas is an extremely poor state (48th and 49th in median household and per capita incomes, respectively) (*Wikipedia, 2015*). And yet, from 2004 to 2013 taxpayers in Arkansas provided about as many dollars to support research in the rich states of California and Massachusetts as were returned to support research in their home state (**Figure S2**). We also provided a similar number of dollars that were shared by the 13 other states that were over funded.

The disposition of research dollars affects every state. At first approximation, each of the 37 states that were under funded subsidized, to varying extents, research in the 15 over-funded states. The net transfer of capital (involving funds that might be expended productively in home states) is large. Based on means of population and per capita funding from 2004 to 2013 (and with the caveats about precision described above), the NIH RPG funding process transferred approximately $4 billion annually from under-funded states to over-funded states. In short, the way that RPG dollars are distributed to individual states has significant impacts on local economies, as well as affecting the diversity and productivity of our national biomedical research enterprise.

The values presented above are estimates based on state populations and RPG funding levels and do not take into account the population density of investigators in each state who are applying for grants. The demographics are important for the following reason.

Eliminating bias will not eliminate disparity

The central thesis of the article is that there are quantifiable biases in the way that RPG funding is allocated to individual states and that the biases should be addressed. But even with equal access to funding (absence of bias), the majority of funding for biomedical research would remain concentrated in a minority of states. This is because past funding differentials have become ingrained in scientific infrastructure and demographics.

Consider, for example, the under-funded state of Arkansas. Based on 2004 to 2013 values, the state contributed about $154 million annually to the RPG funding pool. If there were equal access to funding (interstate parity of success rates and average award sizes), Arkansas would have been awarded about 18% more RPGs and the average size of each award would have been about 20% larger [1]. Under this scenario, the number of Arkansas-derived dollars returned to support research in the state would have increased from about $47 million to about $67 million. Thus, even if geographical bias is eliminated, Arkansas would remain under funded on a per capita basis and it would still send about 57 cents of every RPG tax dollar to subsidize research in other states.

This example suggests that demographics have as great an impact on geographical funding disparities as biases in allocation, which has important implications. First, eliminating bias will not eliminate disparity. Over-funded states with higher population densities of applicants would continue to secure a disproportionate share of total RPG dollars. Second, the fact that funding disparities are driven substantially by demographics prompts thinking on the origins and consequences of demographic differences. For example, to what extent have unbalanced funding decisions in the past shaped current demographics? Are the current demographics in the best interests of the nation's biomedical research enterprise?

While most taxpayers would likely focus on the flow of dollars, scientists and policy makers will consider the issue from perspectives centered more on the research enterprise. On one hand, we should consider advantages and disadvantages of simply accepting the level of disparity conferred by present demographics, as long as scientists in each state have equal access to RPG funding. On the other hand, we should consider advantages and disadvantages of a more balanced distribution of RPG dollars. For example, would the increased diversity and potential productivity gains from a more balanced distribution of funds offset the impacts that this change would have on existing infrastructure? In principle, such scenarios could be evaluated on the basis of empirical data (example below).

Maximizing return on taxpayers' investments

On a per capita basis, all 13 states in the top quartile were over funded (**Table 1**) and that single over-funded quartile received nearly two-thirds of all RPG dollars (**Figure 1A** and **Data S1**). The other three quartiles of states were each under funded (with 37 of 39 states below the national level) (**Table 1**). And yet, the mean productivity in each of the three under-funded

[1] Based on mean values for 2004-2013 (Data S1). Assumes that average annual success rate increases from 14.6% to 17.3% and average award size increases from $327,000 to $393,000 (rounded to nearest thousand); which together would increase total RPG funding to Arkansas by 41.6%.

quartiles was higher or significantly higher than that of the over-funded quartile (**Figure 1E**). This suggests that there might be diminishing marginal returns on RPG investments at the state level, as well as at the level of investigators (*Cook, Grange and Eyre-Walker, 2015; Danthi et al., 2015; Doyle et al., 2015; Lauer et al., 2015; Lorsch, 2015*). It will be interesting to test such inferences further using additional data sets and metrics.

Meanwhile, if one supports the concept of an efficient biomedical research enterprise that maximizes return on taxpayers' investments, then one must consider potential benefits of redistributing funds from the single substantially over-funded quartile of states to the three under-funded quartiles of states. Such considerations for rebalancing should be in addition to eliminating geographical bias because, for reasons described above, eliminating bias would have only an incremental impact on the overall distribution of funds.

Regression analyses of log-transformed data

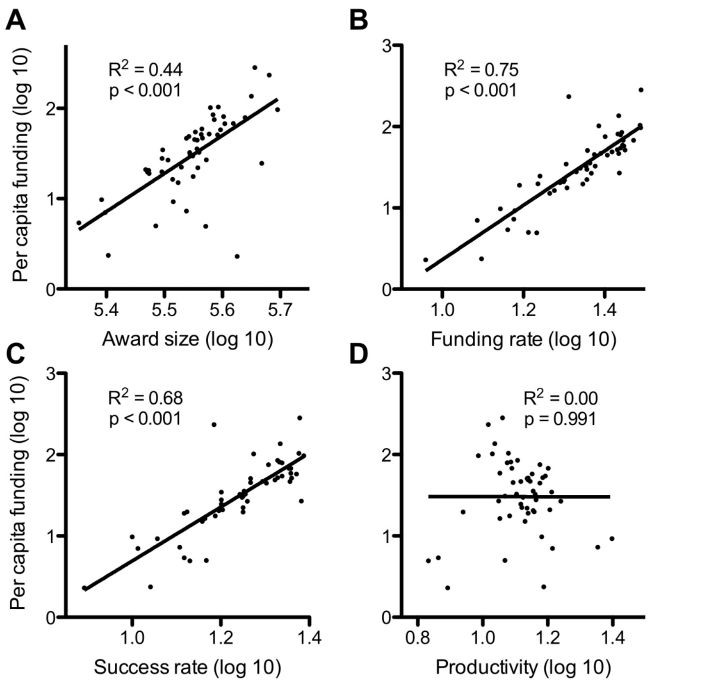

Figure S3. Factors affecting disparities in funding. As in Figure 2, but data were log-transformed prior to analysis. Plots show linear regressions of state per capita RPG funding as a function of: (A) average award size; (B) per investigator funding rate; (C) per application success rate; and (D) scientific productivity. Values are as described in Figure 1 and numerical values by state can be found in Data S1.

It has been suggested that measures of research funding and scientific output can follow a power function (*Fortin and Currie, 2013*), so the linear regressions of non-transformed data (**Figure 2**) were repeated using log-transformed data (**Figure S3**). In two cases the coefficients of determination (R^2) were essentially identical and in two cases higher R^2 values were obtained with the transformed data. This might indicate that power functions better model the impact of success rate and funding rate differences upon per capita funding, although one cannot exclude the possibility that transformation of the data artificially inflates the strengths of association. More to the point, each type of data analysis supports the identical conclusions ($p < 0.001$ for three comparisons, not significant for a fourth).

References

Committee to Evaluate the Experimental Program to Stimulate Competitive Research (EPSCoR) and Similar Federal Agency Programs, Committee on Science, Engineering, and Public Policy, Policy and Global Affairs, National Academy of Sciences, National Academy of Engineering, Institute of Medicine. *The Experimental Program to Stimulate Competitive Research*. Washington DC: National Academies Press (US). ISBN-13: 978-0-309-28828-6 ISBN-10: 0-309-28828-2

Cook I, Grange S, Eyre-Walker A. 2015. Research groups: How big should they be? *PeerJ* 3:e989. doi: 10.7717/peerj.989.

Danthi NS, Wu CO, DiMichele DM, Hoots WK, Lauer MS. 2015. Citation impact of NHLBI R01 grants funded through the American Recovery and Reinvestment Act as compared to R01 grants funded through a standard payline. *Circulation Research* 116:784-788. doi: 10.1161/CIRCRESAHA.116.305894.

Department of Health and Human Services. 2015. HHS FY2015 Budget in Brief. Available at: http://www.hhs.gov/about/budget/fy2015/budget-in-brief/nih/index.html.

Doyle JM, Quinn K, Bodenstein YA, Wu CO, Danthi N, Lauer MS. 2015. Association of percentile ranking with citation impact and productivity in a large cohort of de novo NIMH-funded R01 grants. *Molecular Psychiatry* 20:1030-1036. doi: 10.1038/mp.2015.71.

Fortin, JM, Currie DJ. 2013. Big science vs. little science: how scientific impact scales with funding. *PLoS ONE* 8:e65263. DOI: 10.1371/journal.pone.0065263

Internal Revenue Service. 2015. SOI Tax Stats - Gross Collections, by Type of Tax and State, Fiscal Year - IRS Data Book Table 5. Available at: https://www.irs.gov/uac/SOI-Tax-Stats-Gross-Collections,-by-Type-of-Tax-and-State,-Fiscal-Year-IRS-Data-Book-Table-5

Lauer MS, Danthi NS, Kaltman J, Wu CO. 2015. Predicting productivity returns on investment: Thirty years of peer review, grant funding, and publication of highly cited papers at the National Heart, Lung, and Blood Institute. *Circulation Research* 117:239-243. doi: 10.1161/CIRCRESAHA.115.306830.

Lorsch JR. 2015. Lab size: Is Bigger Better? *iBioMagazine*. Available at: http://www.ibiology.org/ibiomagazine/jon-lorsch-lab-size-is-bigger-better.html

National Institutes of Health. 2014. Table 442-2014, NIH competing research project applications from domestic organizations fiscal years 2004 - 2013.

National Institutes of Health. 2015. Research portfolio online reporting tool (RePORT). Available at: http://report.nih.gov/index.aspx. Accessed on August 13, 2014 and on April 1, 2015.

Rockey S. 2014. Comparing success rates, award rates and funding rates. *RockTalk, NIH Extramural Nexus*. Available at: http://nexus.od.nih.gov/all/2014/03/05/comparing-success-award-funding-rates.

Wikipedia. 2015. List of U.S. states by income, fiscal year 2014. Available at: https://en.wikipedia.org/wiki/List_of_U.S._states_by_income
